# Assay or astray? discrepancies in efanesoctocog alfa monitoring

**DOI:** 10.1016/j.rpth.2026.106994

**Published:** 2026-10-01

**Authors:** Agathe Herb, Jordan Wimmer, Sarra Derbal, Manon Dolt, Léa Pierre, Olivier Feugeas, Laurent Mauvieux, Dominique Desprez, Laurent Sattler

**Affiliations:** 1 Laboratoire d’Hématologie, Unité Hémostase, Hôpitaux Universitaires de Strasbourg, Strasbourg, France; 2 Centre de Ressource et Compétences des Maladies Hémorragiques Congénitales, Hôpitaux Universitaires de Strasbourg, Strasbourg, France

**Keywords:** assay, discrepancies, efanesoctocog alfa, factor VIII, hemophilia A, laboratory, monitoring

## Abstract

**Background:**

Efanesoctocog alfa (EFA) is an ultraextended half-life factor VIII (FVIII) with a unique structure that may influence laboratory monitoring. Although assay discrepancies have been described in spiked samples, data in patient samples are limited.

**Objectives:**

To assess the performance of seven FVIII assays for measuring EFA in patient plasma and compare results with those from spiked samples.

**Methods:**

FVIII activity was measured using four one-stage assays (OSAs) and three chromogenic assays (CSAs) on two analyzers. Assays were calibrated either with human plasma or an EFA-specific calibrator. Measurements were performed in FVIII-deficient plasma spiked with EFA and in samples from patients with hemophilia A treated with EFA. Results were compared with a reference assay (Actin FSL) and expressed as recovery percentages (acceptable range: 80%–120%).

**Results:**

In spiked samples, plasma-calibrated CSA overestimated FVIII activity (+37% to +94%), whereas EFA-calibrated CSA showed good agreement with the reference assay (+2% to +16%). OSA results were generally acceptable, with slight underestimation depending on the reagent. In patient samples, patterns differed markedly: most assays deviated significantly from the reference, and none remained within the acceptable recovery range across the full FVIII spectrum. Compared with spiked samples, discrepancies were more pronounced at later time points and trough levels, where OSA tended to overestimate FVIII activity, whereas EFA-calibrated CSA underestimated activity.

**Conclusion:**

Assay performance for EFA differs between spiked and patient samples, underscoring the limitations of *in vitro* evaluations. No assay provided consistent agreement with the reference assay across the full FVIII range, highlighting the need for cautious interpretation and improved standardization of EFA monitoring in clinical practice.

## Essentials

• Efanesoctocog alfa is a new hemophilia drug that may be measured differently by common lab tests.
• Researchers compared seven tests with a reference assay using patient blood and laboratory-prepared samples.
• Results differed between laboratory-prepared samples and patient samples, especially at later time points.
• No test showed consistent agreement with the reference assay, indicating a need for caution and improved standardization.

## 1 Introduction

Accurate laboratory monitoring of replacement therapy is essential in hemophilia A (HA), particularly in the perioperative setting and during major bleeding. Two assays are used to measure factor VIII (FVIII) activity: one-stage clotting assays (OSA) and chromogenic substrate assays (CSA). The World Federation of Hemophilia recommends using an assay validated for the specific FVIII product administered [1].

Efanesoctocog alfa (EFA; Altuvoct or Altuviiio [Sanofi–Sobi]) is a novel replacement therapy for HA. Its molecular structure is substantially larger than that of native FVIII, consisting of a B-domain-deleted, Fc fusion FVIII linked to the D’D3 domain of von Willebrand factor (VWF), and two XTEN polypeptides [2], [3]. These structural modifications confer a markedly prolonged half-life (37–41 hours), approximately threefold to fourfold longer than that of standard half-life (SHL) and extended half-life (EHL) FVIII products [4]. However, these modifications may also affect laboratory monitoring.

During development, the potency of EFA was most accurately assessed using an Actin FSL-based OSA [2], [5]. This method was ultimately selected for product potency labeling and is currently considered the reference assay [6].

Since then, several studies have evaluated the accuracy of EFA monitoring using OSA and CSA. Pipe et al. performed a multicenter study (*n* = 35) using FVIII-deficient plasma samples spiked with increasing EFA concentrations (5, 20, and 80 IU/dL). Most OSA reagents tended to overestimate EFA activity at 5 IU/dL, whereas some measured it accurately at 20 and 80 IU/dL (e.g., CK-Prest), and Actin FSL showed good accuracy across all concentrations. In contrast, CSA markedly overestimated EFA activity by approximately twofold to threefold [7].

More recently, Reilly-Stitt et al. conducted a large multicenter evaluation including 200 laboratories. Their results suggested that Actin FSL could reliably measure EFA activity at 20, 50, and 100 IU/dL, whereas PTT-A appeared suitable across a broader range (5–100 IU/dL), and CK-Prest performed adequately at 100 IU/dL [8]. This study also confirmed the overestimation of EFA activity observed with CSA. Based on these findings, preliminary recommendations suggest that, for EFA monitoring in the absence of an FVIII mimetic agent (i.e., emicizumab), a human plasma-calibrated OSA using selected reagents (such as Actin FSL, SynthAFax, or CK-Prest) should be used, with local validation [9]. Finally, Nougier et al. further suggested that CSA calibrated with an EFA-specific calibrator may allow accurate EFA monitoring, which may be particularly relevant in patients receiving emicizumab [10].

However, all of the aforementioned studies were conducted using plasma samples spiked with EFA. More recently, Müller et al. reported that assay performance may differ between spiked samples and post-infusion patient samples. In particular, at EFA trough levels, a pronounced difference in CSA/Actin FSL ratios was observed between the two sample types, whereas the ratios were comparable at peak levels [11].

Therefore, evaluating assay performance in plasma from PwHA receiving EFA may better reflect *in vivo* conditions. To our knowledge, such data remain limited. The aim of this study was to assess the performance of seven assays in measuring EFA in patient samples and to determine whether discrepancies exist between patient samples and FVIII-deficient plasma spiked with EFA.

## 2 Methods

This study was approved by the institutional ethics committee (CE-2025-90) and was conducted in accordance with the Declaration of Helsinki. Residual clinical plasma samples were used in accordance with institutional policies. All data were anonymized to ensure patient confidentiality. Samples were obtained from PwHA receiving care at Strasbourg’s Hemophilia Treatment Center and referred to the Hematology Laboratory of Strasbourg University Hospital (Strasbourg, France) for EFA monitoring between June 2025 and March 2026.

Inclusion criteria were patients with severe HA treated with EFA and previously exposed to SHL or EHL FVIII concentrates. A minimum 48-hour washout period from the previous FVIII therapy was required at the time of the switch. Exclusion criteria were insufficient plasma volume, prior treatment with emicizumab, and presence of an FVIII inhibitor.

Blood samples were collected in Vacuette tubes containing 0.109 mol/L (3.2%) sodium citrate (Greiner BioOne). Samples were centrifuged twice at 2500 g for 10 minutes to obtain platelet-poor plasma, aliquoted into polypropylene tubes, and stored at −80 °C until analysis. Frozen aliquots were thawed for 4 minutes in a 37 °C water bath and analyzed within 2 hours.

FVIII activity was measured simultaneously using four OSAs and three CSAs on two analyzers, whenever the available plasma volume was sufficient.

The assays were as follows:

- Actin FSL/Factor VIII-deficient plasma, calibrated with Standard Human Plasma (Siemens) on a CN-3000 analyzer (Sysmex). This assay was used as the reference assay for the comparative assessment in the present study (AFSL OSA).
- PTT-A/Immunodeficient VIII calibrated with Unicalibrator on a STA-R Max 3 analyzer (Diagnostica Stago) (PTT-A OSA).
- CK-Prest/Immunodeficient VIII (Diagnostica Stago) calibrated with Unicalibrator on a STA-R Max 3 (CKP-UNI OSA).
- CK-Prest/Immunodeficient VIII calibrated with a specific EFA calibrator on a STA-R Max 3 (CKP-EFA OSA).
- Factor VIII Chromogenic Assay (Siemens) calibrated with Standard Human Plasma on a CN-3000 (SIE-SHP CSA).
- Factor VIII Chromogenic Assay (Siemens) calibrated with a specific EFA calibrator on a CN-3000 (SIE-EFA CSA).
- Trinichrom Factor VIII:C (Tcoag) calibrated with Unicalibrator on a STA-R Max 3 (TRINI CSA).

The EFA-specific calibrator was prepared by UK NEQAS using congenital FVIII-deficient plasma (from a donor with severe hemophilia A) supplemented with a single batch of Altuvoct. The assigned potency (100 IU/dL) was derived solely from the manufacturer’s labeled potency of the vial. The manufacturer assigns potency using the AFSL OSA.

First, native human congenital FVIII-deficient plasma (Cryopep) was spiked with increasing concentrations of EFA using Altuvoct 250 IU to obtain samples with expected FVIII activities of approximately 10 IU/dL, 35 IU/dL, 80 IU/dL, 100 IU/dL, and 140 IU/dL. These target concentrations were used solely for sample preparation. For assay comparison, FVIII activity measured with AFSL OSA on each individual spiked sample was used as the reference value. FVIII activity was then measured using the other aforementioned assays.

Second, FVIII activity was measured in plasma samples from PwHA treated with EFA to assess the suitability of each assay for EFA monitoring. Blood samples were collected at various time points after EFA infusion: 1 hour (H1), 3 hours (H3), 1 day (D1), 2 days (D2), 4 days (D4), and 7 days (D7).

For each assay, internal quality control (IQC) samples were analyzed at the beginning of each analytical run throughout the study period. For AFSL OSA, IQC results and the corresponding acceptance limits are provided (Supplementary Data S3). All FVIII activity measurements were performed in duplicate, with an acceptance criterion of a coefficient of variation (CV) ≤20%. The final FVIII activity result was defined as the mean of the two measurements. The individual duplicate AFSL OSA results and corresponding CVs for all spiked and patient samples are provided (Supplementary Data S3). For each assay and each sample, FVIII recovery was calculated relative to the reference assay and expressed as a percentage of the corresponding AFSL OSA result, according to the following formula: (measured FVIII activity / AFSL OSA FVIII activity) × 100. An assay was considered compliant when the mean recovery fell within the predefined recovery range (RR) of 80% to 120%. This range was selected a priori as a clinically relevant definition of agreement with the reference assay [12], [13] and was applied consistently across the entire FVIII activity range, as no consensus exists regarding concentration-dependent acceptance criteria for FVIII assay agreement following replacement therapy.

### 2.1 Statistical analysis

Statistical analyses were performed using Prism v6.05 (GraphPad Software). FVIII activity obtained with each assay was compared with the reference assay (AFSL OSA) using the paired Wilcoxon test. A *P* value <.05 was considered statistically significant. Data are presented as mean ± standard deviation (SD) in scatter plots. For box-and-whisker plots, the upper and lower hinges represent the 75th and 25th percentiles (interquartile range, IQR), respectively. Whiskers indicate the minimum and maximum values. The central line represents the median, and the “+” symbol indicates the mean.

## 3 Results

### 3.1 Spiked samples

FVIII activity measured with AFSL OSA in spiked samples ranged from 13 to 162 IU/dL (13, 43, 89, 115 and 162 IU/dL, respectively) and was used as the reference value for subsequent recovery calculations. Compared with AFSL OSA, FVIII activity was consistently overestimated by CSA calibrated with standard plasma (Figure 1). Specifically, SIE-SHP CSA overestimated by +67% to +94%, while TRINI CSA showed overestimation ranging from +37% to +61%. In contrast, SIE-EFA CSA showed good agreement with AFSL OSA, with differences ranging from +2% to +16%.

**Figure 1:**
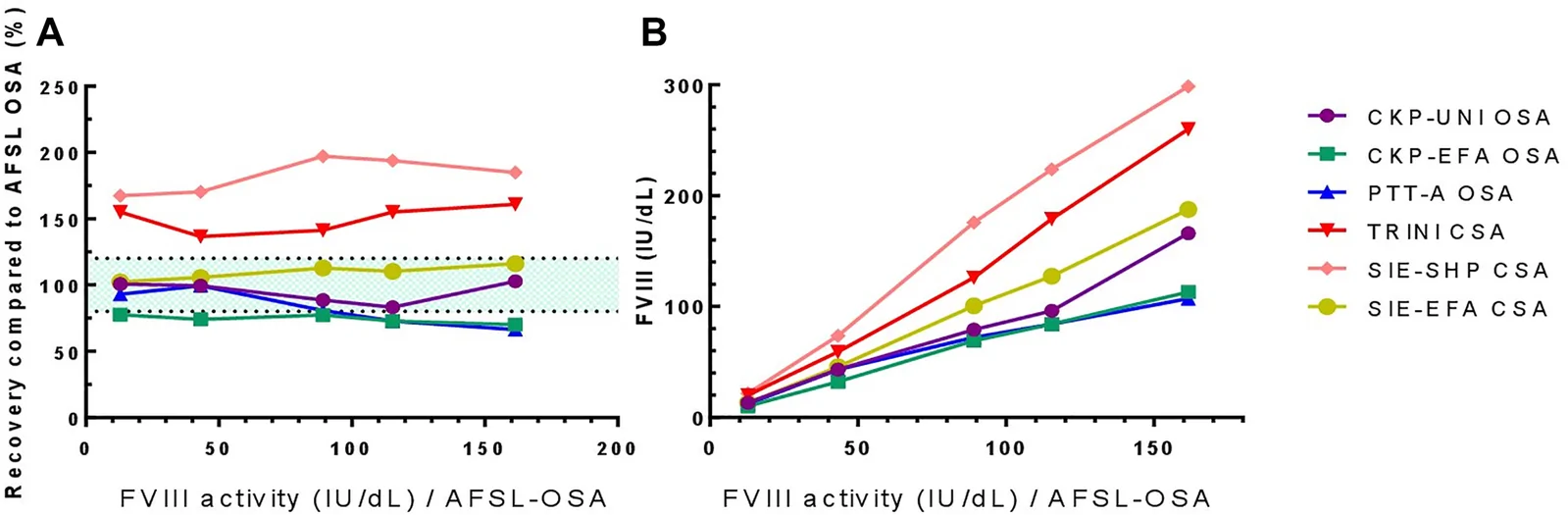
Spiked sample recovery relative to AFSL OSA (A) and FVIII activity as a function of AFSL OSA–measured FVIII activity (B). The green shaded area represents the predefined acceptable recovery range (80%–120%). AFSL, Actin FSL; FVIII, factor VIII; OSA, one-stage assay.

Compared with AFSL OSA, CKP-UNI OSA showed good agreement in spiked samples, with a slight tendency toward underestimation (−17% to +3%). PTT-A OSA showed results within the acceptable RR up to 89 IU/dL (−19% to −7%), but underestimated FVIII activity at higher levels (−34% to −27%). Finally, CKP-EFA OSA slightly underestimated FVIII activity compared with AFSL OSA across all spiked samples (−30% to −23%).

### 3.2 Patient samples

Seventy-three samples from 19 PwHA treated with EFA were included. No results were excluded based on duplicate variability. Mean (min-max) FVIII activity was 72 IU/dL (7–205) with AFSL OSA (*n* = 73), 87 IU/dL (11–229) with CKP-UNI OSA (*n* = 73), 75 IU/dL (8–185) with CKP-EFA OSA (*n* = 73), 73 IU/dL (10–207) with PTT-A OSA (*n* = 63), 95 IU/dL (4–260) with TRINI CSA (*n* = 38), 112 IU/dL (3–366) with SIE-SHP CSA (*n* = 61), and 53 IU/dL (3–178) with SIE-EFA CSA (*n* = 68) (Figure 2; Supplementary Data S1).

**Figure 2:**
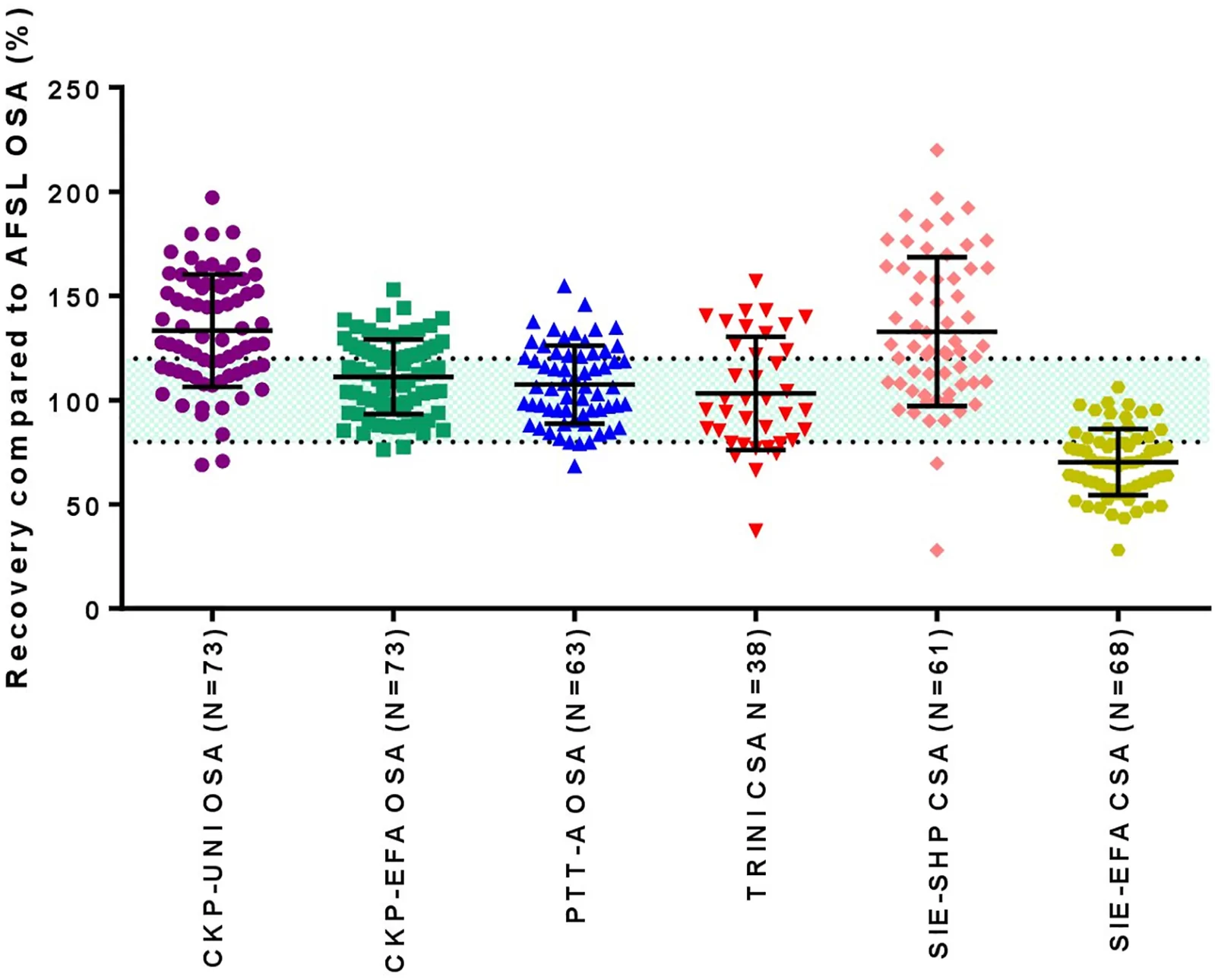
Patient sample recovery relative to AFSL OSA. Green shaded area represents the predefined acceptable recovery range (80%–120%). AFSL, Actin FSL; FVIII, factor VIII; OSA, one-stage assay.

FVIII activity was significantly different from AFSL OSA for CKP-UNI OSA (*P* < .001), CKP-EFA OSA (*P* = .01), SIE-SHP CSA (*P* < .001), and SIE-EFA CSA (*P* < .001). No significant differences were observed between AFSL OSA and PTT-A OSA (*P* = .85) or TRINI CSA (*P* = .34).

In contrast to spiked samples, patient samples showed a tendency toward underestimation with SIE-EFA CSA, whereas CKP-UNI OSA and SIE-SHP CSA exhibited a tendency toward overestimation.

Assay performance varied according to FVIII activity ranges (Figures 3 and 4; Supplementary Data S1). Compared with AFSL OSA, CKP-UNI OSA showed results within the acceptable RR at ≥81 IU/dL, but overestimated values at 41 to 80 IU/dL (+40%), 21 to 40 IU/dL (+51%), and ≤20 IU/dL (+48%). CKP-EFA OSA showed results within the acceptable RR at ≥81 IU/dL and ≤20 IU/dL, but overestimated FVIII activity at 41 to 80 IU/dL (+29%) and 21 to 40 IU/dL (+24%). PTT-A OSA results were within the acceptable RR at ≥21 IU/dL, but showed overestimation at ≤20 IU/dL (+29%).

**Figure 3:**
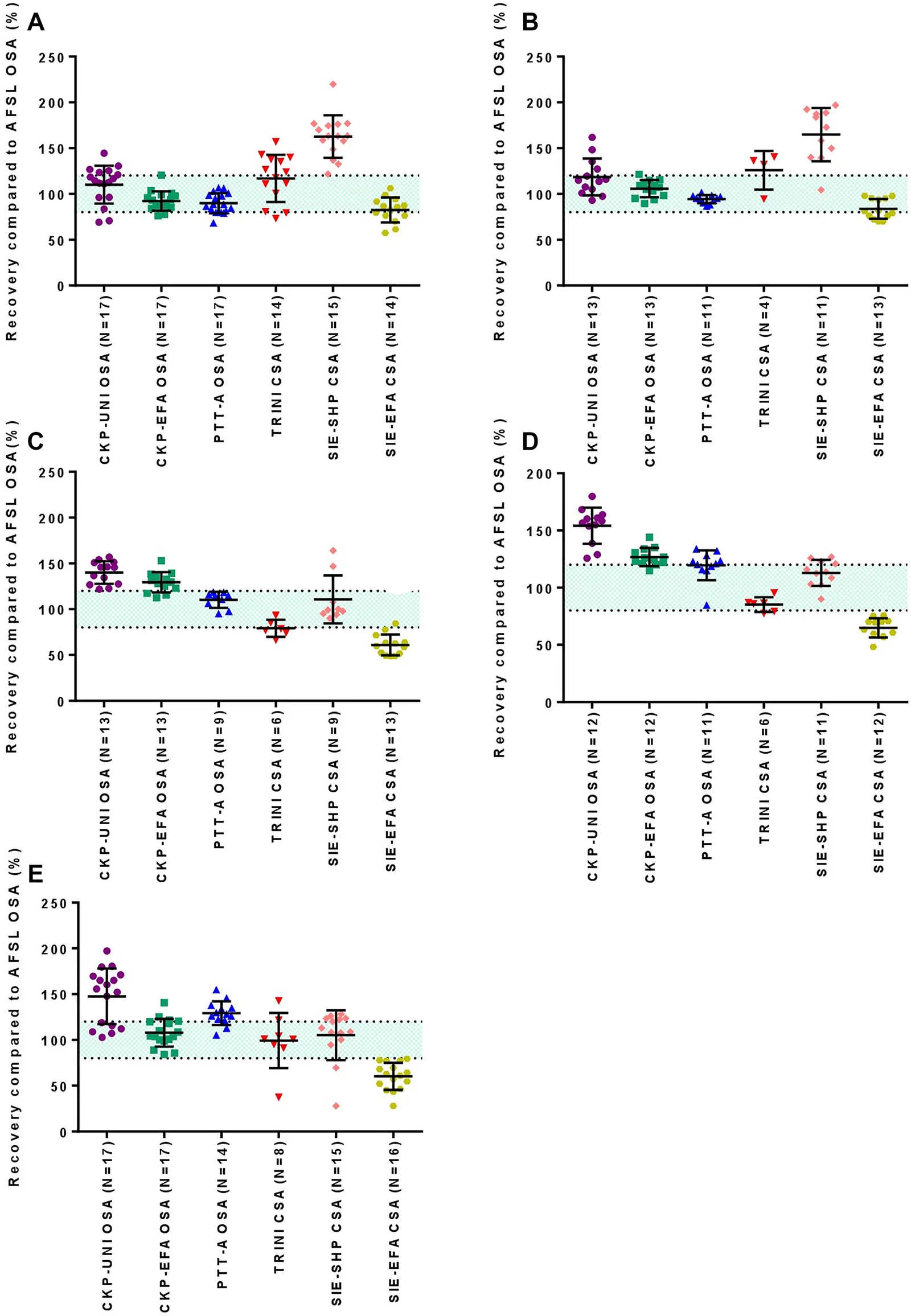
Patient sample recovery relative to AFSL OSA according to FVIII activity ranges measured with AFSL OSA: >120 IU/dL (A), 81–120 IU/dL (B), 41–80 IU/dL (C), 20–41 IU/dL (D), and ≤20 IU/dL (E). Green shaded areas represent the predefined acceptable recovery range (80%–120%). AFSL, Actin FSL; FVIII, factor VIII; OSA, one-stage assay.

**Figure 4:**
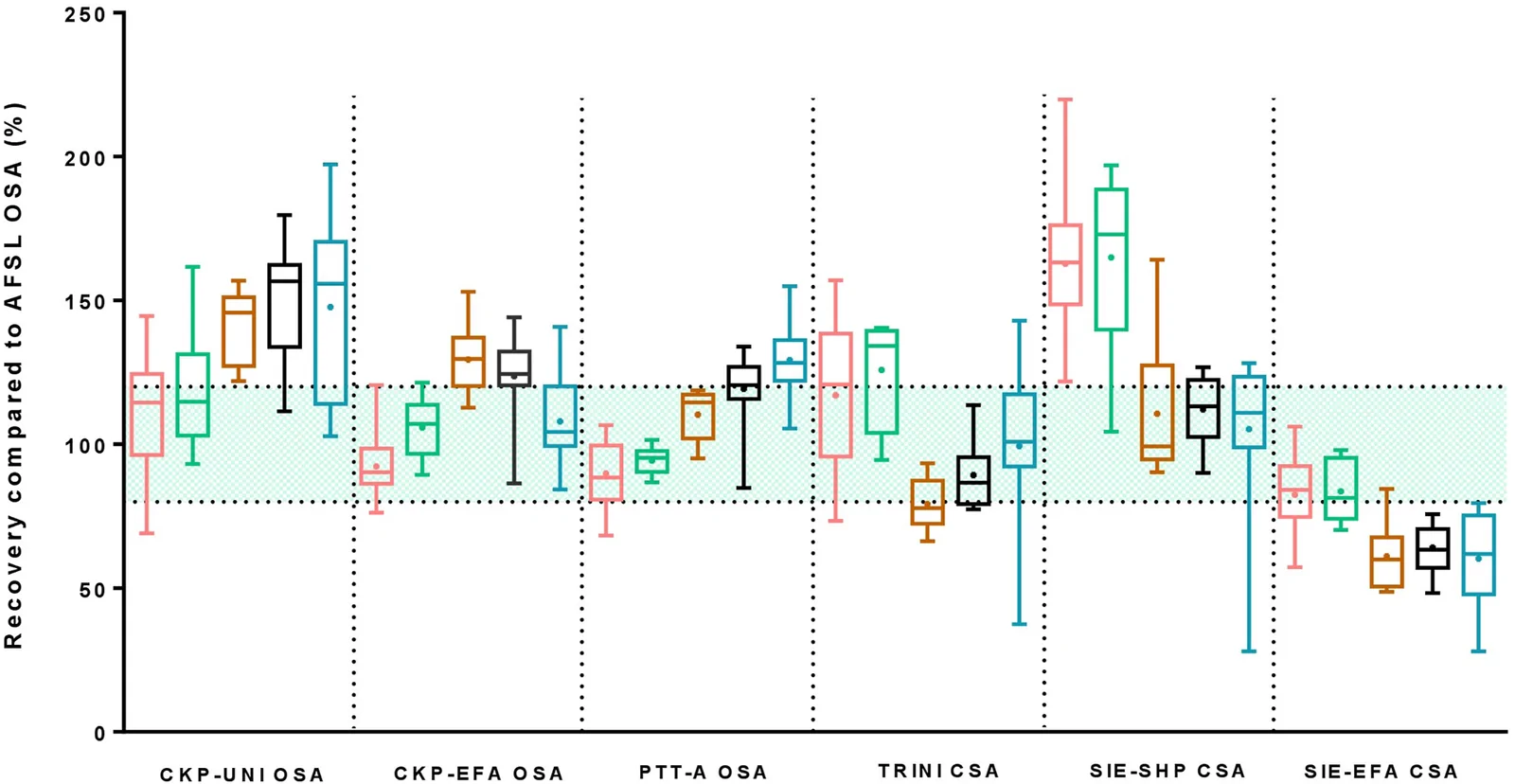
Patient sample recovery relative to AFSL OSA according to FVIII activity ranges measured with AFSL OSA: >120 IU/dL (pink), 81–120 IU/dL (green), 41–80 IU/dL (brown), 21–40 IU/dL (black), and 0–20 IU/dL (blue). The green shaded area represents the predefined acceptable recovery range (80%–120%). AFSL, Actin FSL; FVIII, factor VIII; OSA, one-stage assay.

TRINI CSA yielded mean recoveries within the acceptable RR at FVIII levels >120 IU/dL (despite a tendency toward overestimation) and ≤80 IU/dL, but overestimated FVIII activity at 81 to 120 IU/dL (+26%). Consistent with findings in spiked samples, SIE-SHP CSA markedly overestimated FVIII activity at higher levels (81 to 120 IU/dL: +65%; >120 IU/dL: +63%), whereas results were within the acceptable RR below 80 IU/dL. In contrast, SIE-EFA CSA underestimated FVIII activity at 41 to 80 IU/dL (−39%), 21 to 40 IU/dL (−36%), and ≤20 IU/dL (−40%), while remaining within the acceptable RR above 80 IU/dL.

A similar pattern was observed when stratifying results by time since EFA infusion (Figures 5 and 6; Supplementary Data S1–S2). Compared with AFSL OSA, CKP-UNI OSA showed results within the acceptable RR at peak levels (H1 and H3) but overestimated FVIII activity at later time points (D1: +33%, D2: +25%, D4: +50%, D7: +48%). In contrast to spiked samples, CKP-EFA OSA showed results within the acceptable RR at H1, H3, D1, and D7, with slight overestimation at D2 (+22%) and D4 (+28%). PTT-A OSA remained within the acceptable RR from H1 to D4, with overestimation at D7 (+29%).

**Figure 5:**
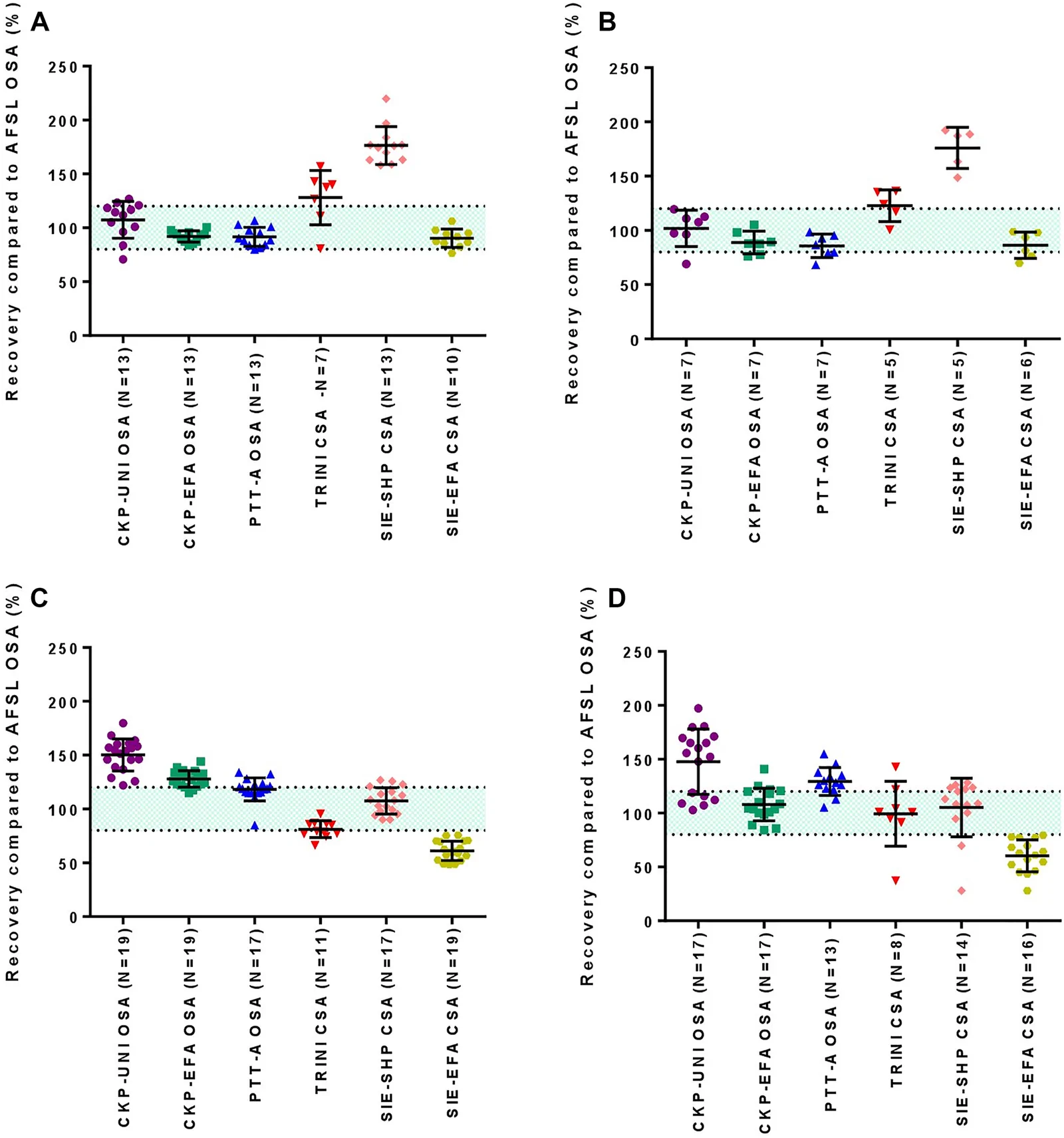
Patient sample recovery relative to AFSL OSA at H1 (A), D1 (B), D4 (C), and D7 (D). Green shaded areas represent the predefined acceptable recovery range (80%–120%). AFSL, Actin FSL; D1, 1 day; D4, 4 days; D7, 7 days; FVIII, factor VIII; H1, 1 hour; OSA, one-stage assay.

**Figure 6:**
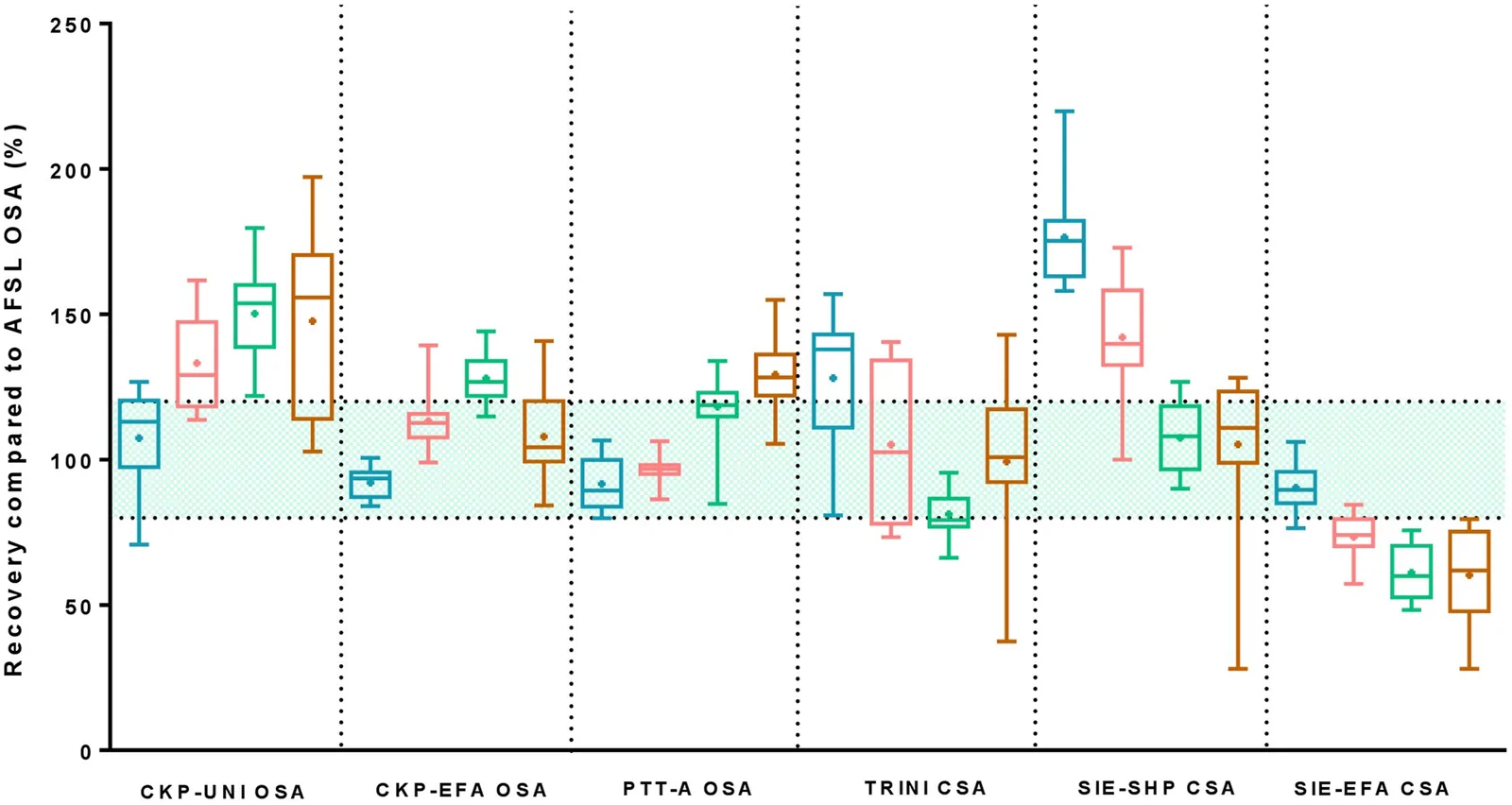
Patient sample recovery relative to AFSL OSA at H1 (blue), D1 (pink), D4 (green), and D7 (brown). Green shaded areas represent the predefined acceptable recovery range (80%–120%). AFSL, Actin FSL; D1, 1 day; D4, 4 days; D7, 7 days; FVIII, factor VIII; H1, 1 hour; OSA, one-stage assay.

TRINI CSA overestimated FVIII activity at peak levels (H1: +28%; H3: +23%) but showed results within the acceptable RR from D1 to D7. SIE-SHP CSA showed marked overestimation at early time points (H1: +77%; H3: +76%; D1: +42%) and acceptable performance thereafter (D2–D7). SIE-EFA CSA showed results within the acceptable RR at H1 and H3 but underestimated FVIII activity at later time points (D1: −27%, D2: −25%, D4: −39%, D7: −40%).

Overall, OSA performance differed between spiked and patient samples. Compared with AFSL OSA, CKP-UNI OSA showed results within the acceptable RR in all spiked samples and in patient samples at FVIII levels >80 IU/dL but overestimated values below this threshold. CKP-EFA OSA underestimated FVIII activity in spiked samples, but tended to overestimate in patient samples at 21 to 80 IU/dL, while remaining within the acceptable RR at >80 IU/dL and ≤20 IU/dL. PTT-A OSA showed results within the acceptable RR in spiked samples up to 89 IU/dL but underestimated at higher levels, whereas patient sample results were within the acceptable RR above 20 IU/dL and overestimated at ≤20 IU/dL.

CSA performance differed even more markedly between spiked and patient samples. Human plasma-calibrated CSA (TRINI CSA and SIE-SHP CSA) showed either acceptable or overestimated results in patient samples, but to a lesser extent than in spiked samples. Compared with AFSL OSA, TRINI CSA consistently overestimated FVIII activity in all spiked samples, but in patient samples remained within the acceptable RR at >120 IU/dL and <81 IU/dL, with overestimation restricted to 81 to 120 IU/dL. SIE-SHP CSA overestimated FVIII activity in all spiked samples and in patient samples above 80 IU/dL, while showing results within the acceptable RR at ≤80 IU/dL. Finally, SIE-EFA CSA yielded results within the acceptable RR in all spiked samples and in patient samples above 80 IU/dL but underestimated values at ≤80 IU/dL.

## 4 Discussion

In this study, we evaluated the performance of four OSAs and three CSAs across two analyzers for monitoring EFA activity, using both spiked FVIII-deficient plasma and postinfusion samples from PwHA. Using AFSL OSA as the reference assay for the comparative assessment of EFA activity [6], our results confirm substantial interassay variability and, importantly, demonstrate marked differences between in vitro and *in vivo* assay performance, with potential implications for clinical monitoring.

In spiked samples, OSA calibrated with either EFA or human plasma provided generally acceptable results, with slight reagent-dependent underestimation. As expected, human plasma-calibrated CSA markedly overestimated EFA activity across all spiked samples (+37% to +94%), whereas EFA-calibrated CSA showed good agreement (+2% to +16%). These findings are consistent with previous studies in spiked samples, which also reported overestimation with human plasma-calibrated CSA and improved accuracy with product-specific calibrators, although recovery was typically assessed against assigned values rather than AFSL OSA. In contrast, we did not observe overestimation with human plasma-calibrated OSA [7], [8], [10]. Moreover, unlike these previous field studies, which evaluated EFA assay performance against labeled potency values, our study assessed inter-assay agreement using AFSL OSA measurements obtained from the same spiked samples as the reference. Therefore, the nominal concentrations used for sample preparation did not influence the comparative assessment of assay performance.

A key finding of our study is that these in vitro observations do not translate directly to patient samples. In our cohort of 73 postinfusion samples, most assays deviated significantly from the reference, and none remained within the predefined acceptable RR across the FVIII spectrum. Compared with spiked samples, discrepancies were more pronounced at later time points (D1 to D4) and at trough levels (D7), indicating time-dependent and concentration-dependent variability.

Indeed, assay behavior varied according to both FVIII activity ranges and time since EFA infusion. OSA tended to overestimate FVIII activity, particularly at trough levels (D7). Plasma-calibrated CSA showed acceptable agreement mainly at later time points (D2–D7), whereas EFA-calibrated CSA tended to underestimate FVIII activity (D1–D7). These findings are consistent with recent observations by Müller et al., who, in a cohort of 26 PwHA assessed using one CSA and one OSA against AFSL OSA, reported concordant CSA/AFSL OSA ratios between spiked and postinfusion samples at peak levels, which became discordant at trough levels. This pattern suggests a time-dependent differential assay behavior between *in vitro* and *in vivo* conditions [11].

These discrepancies likely reflect the complex *in vivo* behavior of EFA. Unlike conventional FVIII concentrates, EFA is a structurally modified molecule combining a B-domain-deleted FVIII with an Fc fragment, the D’D3 domain of VWF, and XTEN polypeptides, resulting in increased molecular size and prolonged half-life [2], [3]. While this design enables independence from endogenous VWF, it may also lead to heterogeneous circulating forms not reproduced in spiked plasma, thereby affecting assay responses, as previously hypothesized [11].

Mechanistically, differences between OSAs and CSAs may further contribute to variability. OSAs rely on clot formation kinetics following contact activation, whereas CSAs measure factor Xa generation within a defined enzymatic system [13]. In addition, *in vivo* processes such as proteolytic cleavage or conformational changes may alter EFA activity over time, consistent with the increasing discrepancies observed at later time points [11], [14].

Our observations may have important clinical implications. Although PTT-A OSA and TRINI CSA did not differ significantly from AFSL OSA overall, none of the evaluated assays remained consistently within the predefined acceptable RR across the full FVIII range. Most assays, including OSA and human plasma-calibrated CSA, tended to overestimate EFA activity, whereas EFA-calibrated CSA tended to underestimate it. This is particularly relevant for clinical decision-making, as reliable FVIII activity assessment is essential for prophylaxis optimization and perioperative management [1]. During surgery, overestimation of FVIII activity may lead to underdosing and an increased risk of bleeding, whereas underestimation may result in unnecessary dose escalation and a higher thrombotic risk. These issues are especially critical in specific contexts such as patients receiving emicizumab, in whom OSAs are unreliable and bovine-based CSAs are required [15]. Although EFA-calibrated CSA may represent a suitable option [10], our results suggest that underestimation at normal to low FVIII levels warrants caution.

Our findings may also challenge current assay selection strategies, which are largely based on spiked plasma studies. While the use of selected OSA reagents or product-specific calibrators has been proposed, these approaches may not fully capture the biological complexity of EFA *in vivo* [7], [8], [9], [10], [16]. Laboratories should therefore carefully select and locally validate assays for EFA monitoring.

This study has several limitations. It was conducted in a single center and included a limited number of patients. No investigations of *in vivo* EFA behavior were performed to explain the observed discrepancies. Importantly, all comparisons were based on the local AFSL OSA results, which were used as the reference assay for both spiked and patient samples. Therefore, the observed recoveries and the conclusions regarding the relative performance of the other assays are dependent on the accuracy and analytical performance of the AFSL OSA under the conditions of our study. Although duplicate measurements, the corresponding CVs, and IQC data are provided in the supplementary data (Supplementary Data S3), a formal measurement uncertainty was not established for this study-specific implementation of the AFSL OSA. In addition, the AFSL OSA was not included in an independent external quality assessment or proficiency testing program during the study period because it was not used as a routine clinical assay. These limitations should be considered when interpreting the results and emphasize that our findings represent comparative assay performance relative to the local AFSL OSA rather than an absolute assessment of FVIII activity accuracy. Nevertheless, our study provides clinically relevant data based on real-world patient samples, complementing existing *in vitro* evidence.

Further research is warranted to better characterize the *in vivo* behavior of EFA. In particular, future studies should investigate whether global coagulation assays, such as thrombin generation assays, could provide complementary information on the *in vivo* hemostatic activity of EFA.

## 5 Conclusion

EFA monitoring is assay-dependent and differs substantially between spiked and patient samples when assessed relative to AFSL OSA. The observed discrepancies may have clinically relevant consequences for treatment decisions. These findings should nevertheless be interpreted in light of the fact that AFSL OSA served as the local reference assay, and therefore reflect comparative assay performance rather than absolute accuracy. They underscore the need for cautious interpretation of FVIII activity in patients treated with EFA and suggest that assay selection strategies based solely on *in vitro* data may be insufficient. Further studies are required to optimize and standardize EFA monitoring in clinical practice.
