# Supplementary material for: Assay or astray? discrepancies in efanesoctocog alfa monitoring
Source: Res Pract Thromb Haemost. 2026 Oct 1;10(7):106994. doi: 10.1016/j.rpth.2026.106994 (PMC13717027; doi:10.1016/j.rpth.2026.106994)
Supplement: Supplementary file 1 [file mmc3.pdf]

SPIKED SAMPLES

Expected FVIII activity	FVIII - AFSL 1	FVIII - AFSL 2	Mean	CV(%)
140	166,1	156,8	161,5	5,6
100	111,3	119,5	115,4	-7,4
80	85,9	92,4	89,2	-7,6
35	45,1	41,3	43,2	8,4
10	13,7	12,1	12,9	11,7

PATIENTS SAMPLES

SAMPLES	EFA INFUSION	FVIII - AFSL 1	FVIII - AFSL 2	Mean	CV(%)
AC-D1-1	D1	88,0	80,0	84,0	9,1
AC-D4-1	D4	36,0	31,0	33,5	13,9
AC-D7-1	D7	12,5	10,5	11,5	16,0
AC-H1-1	H1	123,4	119,7	121,6	3,0
AC-H1-2	H1	129,2	120,8	125,0	6,5
AC-H3-1	H3	115,1	110,9	113,0	3,6
AC-H3-2	H3	116,8	124,2	120,5	-6,3
AM-D4-1	D4	49,3	50,1	49,7	-1,6
AM-D7-1	D7	15,6	16,3	16,0	-4,5
AR-D1-1	D1	84,1	87,4	85,8	-3,9
AR-D4-1	D4	23,0	22,5	22,8	2,2
AR-D7-1	D7	10,7	10,5	10,6	1,9
AR-H1-1	H1	126,7	115,4	121,1	8,9
AS-D1-1	D1	95,1	91,4	93,3	3,9
AS-D2-1	D2	121,0	115,1	118,1	4,9
AS-D7-1	D7	7,4	8,0	7,7	-8,1
AS-D7-2	D7	10,2	8,8	9,5	13,7
AS-D7-3	D7	13,3	14,7	14,0	-10,5
BD-D1-1	D1	137,4	131,2	134,3	4,5
BD-D4-1	D4	37,9	38,3	38,1	-1,1
BD-D7-1	D7	10,1	11,7	10,9	-15,8
BD-H1-1	H1	184,0	193,1	188,6	-4,9
BL-D1-1	D1	104,6	98,9	101,8	5,4
BL-H1-1	H1	159,5	166,3	162,9	-4,3
ER-D7-1	D7	13,1	11,7	12,4	10,7
FM-D4-1	D4	30,8	27,3	29,1	11,4
FM-D4-2	D4	32,9	33,7	33,3	-2,4
JH-D1-1	D1	117,7	125,2	121,5	-6,4
JH-D4-1	D4	43,0	44,0	43,5	-2,3
JH-D7-1	D7	12,3	10,7	11,5	13,0
JH-H1-1	H1	154,8	162,1	158,5	-4,7
JH-H3-1	H3	162,0	167,0	164,5	-3,1
JP-D2-1	D2	83,2	77,8	80,5	6,5
JP-D2-2	D2	85,1	86,7	85,9	-1,9
JP-D4-1	D4	29,6	29,4	29,5	0,7
JP-D4-2	D4	34,0	33,1	33,6	2,6
JP-D7-1	D7	6,8	7,4	7,1	-8,8
JP-H1-1	H1	162,9	160,9	161,9	1,2

JP-H1-2	H1	162,9	170,0	166,5	-4,4
JR-D1-1	D1	103,2	104,5	103,9	-1,3
JR-D4-1	D4	44,4	45,5	45,0	-2,5
JR-D7-1	D7	14,7	15,7	15,2	-6,8
JR-H1-1	H1	132,1	132,2	132,2	-0,1
JR-H3-1	H3	154,3	151,4	152,9	1,9
LE-D1-1	D1	62,0	60,0	61,0	3,2
LE-D3-1	D3	49,1	46,2	47,7	5,9
LE-D7-1	D7	10,0	11,4	10,7	-14,0
MN-D7-1	D7	14,1	15,3	14,7	-8,5
OB-D1-1	D1	148,0	159,0	153,5	-7,4
OB-D4-1	D4	47,0	45,0	46,0	4,3
OB-D7-1	D7	12,0	11,0	11,5	8,3
OB-H1-1	H1	202,0	208,0	205,0	-3,0
OB-H3-1	H3	199,0	206,0	202,5	-3,5
RH-D1-1	D1	62,1	62,1	62,1	0,0
RH-D4-1	D4	22,5	23,0	22,8	-2,2
RH-D7-1	D7	6,7	7,7	7,2	-14,9
RH-H1-1	H1	94,7	95,7	95,2	-1,1
RH-H3-1	H3	84,9	90,1	87,5	-6,1
SL-D1-1	D1	76,6	83,1	79,9	-8,5
SL-D4-1	D4	29,9	30,4	30,2	-1,7
SL-D7-1	D7	11,1	11,3	11,2	-1,8
SL-H1-1	H1	115,4	110,6	113,0	4,2
SL-H3-1	H3	98,7	103,9	101,3	-5,3
SM-D1-1	D1	105,0	106,1	105,6	-1,0
SM-D4-1	D4	31,4	29,9	30,7	4,8
SM-D7-1	D7	8,5	8,2	8,4	3,5
VV-D4-1	D4	33,0	30,1	31,6	8,8
VV-D4-2	D4	34,1	35,8	35,0	-5,0
VV-D4-3	D4	59,4	56,1	57,8	5,6
VV-D4-4	D4	63,0	64,2	63,6	-1,9
VV-D4-5	D4	68,0	63,1	65,6	7,2
YA-D2-1	D2	56,2	59,4	57,8	-5,7
YA-D2-2	D2	62,7	66,7	64,7	-6,4

VIII activity - AFSL internal quality control

Date	Control Plasma P (Siemens) Batch 556756 Range: 23-39 IU/dL	Control Plasma N (Siemens) Batch 507955 Range: 75 - 113 IU/dL
4-Dec-25	32,1	81,7
8-Jan-26	32,7	88,3
22-Jan-26	28	81,6
11-Mar-26	31,9	85,7
