# Supplementary material for: Assay or astray? discrepancies in efanesoctocog alfa monitoring
Source: Res Pract Thromb Haemost. 2026 Oct 1;10(7):106994. doi: 10.1016/j.rpth.2026.106994 (PMC13717027; doi:10.1016/j.rpth.2026.106994)
Supplement: Supplementary file 2 [file mmc1.docx]

**Supplementary Table 1**: Patient samples FVIII activity and recovery relative to AFSL OSA

|  | AFSL OSA | CKP-UNI OSA | CKP-EFA OSA | PTT-A OSA | TRINI CSA | SIE-SHP CSA | SIE-EFA CSA |
| --- | --- | --- | --- | --- | --- | --- | --- |
| N | 73 | 73 | 73 | 63 | 38 | 61 | 68 |
| Mean (IU/dL) (min-max) | 72 (7 – 205) | 87 (11 – 229) | 75 (8 – 185) | 73 (10 – 207) | 95 (4 – 260) | 112 (3 – 366) | 53 (3 – 178) |
| Mean recovery (%) (min-max) |  | 133 (69 – 197) | 111 (76 – 153) | 107 (68 – 155) | 103 (37 – 157) | 133 (28 – 220) | 70 (28 – 106) |

|  | | AFSL OSA | CKP-UNI OSA | CKP-EFA OSA | PTT-A OSA | TRINI CSA | SIE-SHP CSA | SIE-EFA CSA |
| --- | --- | --- | --- | --- | --- | --- | --- | --- |
| FVIII > 120 IU/dL | N | 17 | 17 | 17 | 17 | 14 | 15 | 14 |
|  | Mean (IU/dL) (min-max) | 152 (121 – 205) | 168 (105 – 229) | 140 (105 – 185) | 139 (96 – 207) | 185 (89 – 260) | 253 (167 – 366) | 124 (75 – 178) |
|  | Mean recovery (%) (min-max) |  | 110 (69 – 145) | 92 (76 – 121) | 90 (68 – 107) | 117 (73 – 157) | 163 (122 – 220) | 82 (57 – 106) |
| FVIII 81 – 120 IU/dL | N | 13 | 13 | 11 | 4 | 11 | 13 | 13 |
|  | Mean (IU/dL) (min-max) | 98 (81 – 118) | 117 (96 – 168) | 104 (90 – 122) | 92 (82 – 102) | 122 (81 – 154) | 163 (90 – 223) | 83 (59 – 113) |
|  | Mean recovery (%) (min-max) |  | 118 (93 – 162) | 106 (89 – 121) | 94 (87 – 102) | 126 (95 – 141) | 165 (104 – 197) | 84 (70 – 98) |
| FVIII 41 – 80 IU/dL | N | 13 | 13 | 13 | 9 | 6 | 9 | 13 |
|  | Mean (IU/dL) (min-max) | 57 (44 – 80) | 80 (64 – 102) | 74 (54 – 93) | 63 (50 - 78) | 44 (31 - 57) | 65 (42 - 131) | 36 (23 – 68) |
|  | Mean recovery (%) (min-max) |  | 140 (122 – 157) | 129 (113 – 153) | 110 (95 – 119) | 80 (66 – 93) | 111 (90 – 164) | 61 (49 – 85) |
| FVIII 21 – 40 IU/dL | N | 12 | 12 | 12 | 11 | 6 | 11 | 12 |
|  | Mean (IU/dL) (min-max) | 31 (23 – 38) | 47 (35 – 59) | 39 (28 – 48) | 36 (26 – 47) | 28 (25 – 33) | 34 (26 – 43) | 20 (14 – 29) |
|  | Mean recovery (%) (min-max) |  | 151 (111 – 180) | 124 (86 – 144) | 120 (85 – 134) | 85 (77 – 114) | 113 (90 – 127) | 64 (48 – 76) |
| FVIII ≤ 20 IU/dL | N | 17 | 17 | 17 | 14 | 8 | 14 | 16 |
|  | Mean (IU/dL) (min-max) | 11 (7 – 16) | 16 (11 – 26) | 12 (8 – 19) | 15 (10 – 26) | 11 (4 – 14) | 11 (3 – 15) | 7 (3 – 10) |
|  | Mean recovery (%) (min-max) |  | 148 (103 – 197) | 108 (84 – 141) | 129 (105 – 155) | 99 (37 – 143) | 105 (28 – 128) | 60 (28 – 80) |

**Supplementary Table 2**: Patient samples FVIII activity and recovery relative to AFSL OSA according to FVIII activity ranges measured with AFSL OSA

|  | | AFSL OSA | CKP-UNI OSA | CKP-EFA OSA | PTT-A OSA | TRINI CSA | SIE-SHP CSA | SIE-EFA CSA |
| --- | --- | --- | --- | --- | --- | --- | --- | --- |
| H1 | N | 12 | 12 | 12 | 12 | 7 | 12 | 10 |
|  | Mean (IU/dL) (min-max) | 146 (95 – 205) | 158 (100 – 229) | 134 (91 – 185) | 135 (86 – 207) | 209 (101 – 260) | 257 (175 – 366) | 128 (91 – 178) |
|  | Mean recovery (%) (min-max) |  | 107 (71 – 127) | 92 (84 – 101) | 92 (80 – 107) | 128 (81 – 157) | 177 (158 -220) | 90 (76 – 106) |
| H3 | N | 7 | 7 | 7 | 5 | 5 | 6 | 7 |
|  | Mean (IU/dL) (min-max) | 135 (88 – 203) | 137 (105 – 228) | 117 (92 – 178) | 115 (84 – 200) | 183 (154 – 238) | 229 (165 – 331) | 110 (82 – 155) |
|  | Mean recovery (%) (min-max) |  | 102 (69 - 119) | 89 (76 – 105) | 86 (68 – 99) | 123 (101 – 136) | 176 (149 – 192) | 86 (70 – 99) |
| D1 | N | 12 | 12 | 12 | 11 | 6 | 11 | 12 |
|  | Mean (IU/dL) (min-max) | 99 (61 – 154) | 131 (84 – 194) | 111 (70 – 162) | 96 (58 – 163) | 112 (57 – 150) | 141 (61 – 187) | 72 (44 – 107) |
|  | Mean recovery (%) (min-max) |  | 133 (114 – 162) | 113 (99 – 139) | 97 (86 – 106) | 105 (73 – 141) | 142 (100 – 173) | 73 (57 – 85) |
| D2 | N | 5 | 5 | 5 | 2 | 1 | 2 | 4 |
|  | Mean (IU/dL) (min-max) | 81 (58 – 118) | 99 (71 – 117) | 98 (74 – 122) | 87 (87 – 87) | 81 | 99 (90 – 109) | 64 (37 – 113) |
|  | Mean recovery (%) (min-max) |  | 125 (93 – 145) | 122 (103 – 134) | 105 (102 – 108) | 95 | 120 (104 – 135) | 75 (64 – 95) |
| D4 | N | 19 | 19 | 19 | 17 | 11 | 17 | 19 |
|  | Mean (IU/dL) (min-max) | 39 (23 – 66) | 58 (35 – 96) | 50 (28 – 89) | 45 (26 – 76) | 34 (25 – 56) | 40 (26 – 64) | 23 (14 – 39) |
|  | Mean recovery (%) (min-max) |  | 150 (122 – 180) | 128 (115 – 144) | 118 (85 – 134) | 81 (66 – 96) | 108 (90 – 127) | 61 (48 – 76) |
| D7 | N | 17 | 17 | 17 | 14 | 8 | 14 | 16 |
|  | Mean (IU/dL) (min-max) | 11 (7 -16) | 16 (11 – 26) | 12 (8 – 19) | 15 (10 – 26) | 11 (4 – 14) | 11 (3 – 15) | 7 (3 – 10) |
|  | Mean recovery (%) (min-max) |  | 148 (103 – 197) | 108 (84 – 141) | 129 (105 – 155) | 99 (37 – 143) | 105 (28 – 128) | 60 (28 – 80) |

**Supplementary Table 3**: Patient samples FVIII activity and recovery relative to AFSL OSA at H1, H3, D1, D2, D4 and D7.
