# Supplementary material for: Assay or astray? discrepancies in efanesoctocog alfa monitoring
Source: Res Pract Thromb Haemost. 2026 Oct 1;10(7):106994. doi: 10.1016/j.rpth.2026.106994 (PMC13717027; doi:10.1016/j.rpth.2026.106994)
Supplement: Supplementary file 3 [file mmc2.docx]

**Supplementary Figure 1**: Patient samples recovery relative to AFSL OSA at H2. Green shaded area represents the predefined acceptable recovery range (80–120%).

**Supplementary Figure 2**: Patient samples recovery relative to AFSL OSA at D2. Green shaded area represents the predefined acceptable recovery range (80–120%).
